# Enasidenib, an inhibitor of mutant IDH2 proteins, induces durable remissions in older patients with newly diagnosed acute myeloid leukemia

**DOI:** 10.1038/s41375-019-0472-2

**Published:** 2019-04-09

**Authors:** Daniel A. Pollyea, Martin S. Tallman, Stéphane de Botton, Hagop M. Kantarjian, Robert Collins, Anthony S. Stein, Mark G. Frattini, Qiang Xu, Alessandra Tosolini, Wendy L. See, Kyle J. MacBeth, Samuel V. Agresta, Eyal C. Attar, Courtney D. DiNardo, Eytan M. Stein

**Affiliations:** 1 Division of Hematology, University of Colorado School of Medicine, Aurora, CO, USA; 2 Leukemia Service, Department of Medicine, Memorial Sloan Kettering Cancer Center, New York, NY, USA; 3 Université Paris-Sud, Université Paris-Saclay, Le Kremlin-Bicêtre, France; 4 Gustave Roussy, Département d’hématologie et Département d’innovation Thérapeutique, F-94805 Villejuif, France; 5 The University of Texas MD Anderson Cancer Center, Houston, TX, USA; 6 UT Southwestern Medical Center, Dallas, TX, USA; 7 City of Hope Comprehensive Cancer Center, Duarte, CA, USA; 8 Celgene Corporation, Summit, NJ, USA; 9 Agios Pharmaceuticals, Inc., Cambridge, MA, USA; 10 Weill Cornell Medical College, New York, NY, USA

## Abstract

Older adults with acute myeloid leukemia (AML) who are not fit for standard chemotherapy historically have poor outcomes. Approximately 12–15% of older patients with AML harbor *isocitrate dehydrogenase 2* (*IDH2*) gene mutations. Enasidenib is an oral inhibitor of mutant *IDH2* proteins. Among 39 patients with newly diagnosed mutant-*IDH2* AML who received enasidenib monotherapy in this phase I/II trial, median age was 77 years (range 58–87) and 23 patients (59%) had had an antecedent hematologic disorder. The median number of enasidenib treatment cycles was 6.0 (range 1–35). The most common treatment-related adverse events were indirect hyperbilirubinemia (31%), nausea (23%), and fatigue, decreased appetite, and rash (18% each). Treatment-related grade 3–4 cytopenias were reported for eight patients (21%); there was no treatment-related grade 3–4 infections. Twelve patients achieved a response (overall response rate 30.8% [95% CI 17.0%, 47.6%]), including seven patients (18%) who attained complete remission. At a median follow-up of 8.4 months, the median duration of any response was not reached (NR). Median overall survival for all patients was 11.3 months (95% CI 5.7, 15.1), and was NR for responders. Oral, outpatient targeted treatment with enasidenib may benefit older adults with newly diagnosed mutant-*IDH2* AML who are not candidates for cytotoxic regimens.

## Introduction

Acute myeloid leukemia (AML) primarily affects older adults; the median age at diagnosis is 68 years and its incidence increases with age [1]. Older patients with AML are more likely to have clinical features associated with therapeutic resistance, such as treatment-related disease, adverse cytogenetic or molecular abnormalities, and antecedent hematologic disorders [2]. Moreover, older patients are less likely to tolerate intensive chemotherapy regimens, and those who do receive them are at relatively high risk of treatment-related mortality [2, 3]. In a retrospective, population-based study of 5480 older patients with newly diagnosed AML (median age 78 years), more than one-half of patients (53%) who received treatment died within 60 days of AML diagnosis, with a 60-day mortality rate of 36% in patients who received intensive chemotherapy and 19% in patients treated with a hypomethylating agent (HMA) [2]. Older patients with untreated AML who are not candidates for standard induction therapy due to advanced age, poor performance status, comorbidities, poor-risk cytogenetic or molecular features, or other factors, pose a distinct therapeutic challenge. Accordingly, older patients often receive no leukemia-directed therapy. A retrospective cohort analysis of 8336 patients diagnosed with AML between 2000 and 2009 in the United States Surveillance, Epidemiology, and End Results (SEER) Medicare database indicated that only 40% of patients aged >66 years received active treatment within 3 months of diagnosis [4]. Median overall survival (OS) for older AML patients who did not receive therapy was 2 months, and for those who received available lower-intensity strategies was ~6 months [2–4]. Older patients treated without an allogeneic stem cell transplantation had a 10-year OS of only 2.4% [5].

AML is associated with recurrent chromosomal abnormalities and somatic mutations [6–8]. Mutations in *isocitrate dehydrogenase 2* (*IDH2*) genes, at active site arginine residues in codons R140 and R172, are among the most frequent mutations in AML [6, 7]. *IDH2* mutations occur in ~12–15% of patients with AML [9], with higher frequencies in older patients; mutation analyses in a subgroup of patients aged ≥65 years with newly diagnosed AML in a phase III study of azacitidine showed 23% of patients had an *IDH2* mutation at study entry [10].

IDH2 proteins play a central role in the citric acid cycle, catalyzing the conversion of isocitrate to α-ketoglutarate (α-KG). Mutant IDH proteins have neomorphic activity, reducing α-KG to the oncometabolite, (R)-2-hydroxyglutarate (2-HG) [11]. Elevated concentrations of 2-HG, which are observed in mutant-*IDH2* malignancies, competitively inhibit α-KG-dependent epigenetic regulators, including histone demethylases and DNA methylcytosine dioxygenases of the TET family of proteins, leading to hypermethylation of histones and DNA and blocked cellular differentiation [12–14].

Enasidenib (IDHIFA®, AG-221; Celgene Corporation, Summit, NJ, USA) is an oral inhibitor of mutant-*IDH2* proteins approved in the United States for the treatment of adult patients with mutant-*IDH2* relapsed or refractory (R/R) AML. In preclinical and clinical studies, enasidenib was shown to decrease intracellular 2-HG to normal levels and induce differentiation of mutant-*IDH2* immature myeloid precursor and progenitor cells in responding patients [15–17]. In a pivotal phase I/II study of enasidenib monotherapy (AG221-C-001; NCT01915498), overall response rate (ORR) in patients with mutant-*IDH2* R/R AML was 38.8%, with a complete remission (CR) rate of 19.6%. The median OS among all patients with R/R AML was 8.8 months [18]. The study also allowed enrollment of a limited number of patients with newly diagnosed mutant-*IDH2* AML who were not candidates for standard chemotherapy. This is the first report of clinical outcomes with enasidenib monotherapy in older patients with newly diagnosed AML.

## Methods

This multicenter, open-label, single-arm study (NCT 01915498) enrolled patients with advanced hematologic malignancies harboring an *IDH2* mutation. Study design and methods have been reported in detail elsewhere [15, 16]. The current prospective analyses include patients with previously untreated mutant-*IDH2* AML who were not candidates for standard AML treatments and who had Eastern Cooperative Oncology Group (ECOG) performance status scores of 0–2 at study entry. Presence of *IDH2* mutations was assessed locally at the clinical site. The study protocol was approved by relevant ethics committees and/or institutional review boards at all participating sites. Written informed consent was provided by all patients before participation in the study.

Patients who enrolled in the dose-escalation portion of the study received total enasidenib doses of 50–650 mg/day. Enasidenib was administered orally in continuous 28-day treatment cycles. Bone marrow biopsies and/or aspirates and peripheral blood were collected at screening, on day 1 of treatment cycle 2, every 28 days for the next 12 months, and then every 56 days thereafter during enasidenib treatment.

Safety was assessed by investigator-reported treatment-emergent adverse events (TEAEs), graded according to the National Cancer Institute Common Terminology Criteria for Adverse Events (NCI-CTCAE) version 4.03. TEAEs were defined as any adverse events that began or worsened on or after the start of enasidenib treatment through 28 days after the last dose.

Response was assessed per modified IWG 2003 response criteria for AML [19]. ORR included CR, CR with incomplete hematologic or platelet recovery (CRi/CRp), morphologic leukemia-free state (MLFS), and partial remission (PR). Stable disease was defined as the absence of an IWG-defined hematologic response with no evidence of disease progression, sustained for at least 56 consecutive days.

OS was defined as the time from first enasidenib dose to death from any cause. Event-free survival (EFS) comprised the interval between first enasidenib dose and AML relapse (≥5% bone marrow blasts, reappearance of blasts in blood, or development of extramedullary disease), disease progression, or death.

Transfusion dependence at baseline was defined as having received at least 1 unit of transfused platelets or red blood cells (RBCs) within the first 4 weeks before beginning enasidenib and the first 4 weeks after the first dose in phase I of the study, and as requiring a transfusion during the 8 weeks before beginning enasidenib treatment in study phase II. Transfusion independence was defined as no RBC or no platelet transfusion for at least 56 consecutive days on study.

Correlations between translational endpoints and response were assessed for patients with available baseline data and at least one post-baseline efficacy assessment. Total 2-HG concentrations before and during treatment were evaluated by liquid chromatography tandem mass spectrometry according to an analytically validated method. Next-generation sequencing of DNA isolated from bone marrow or peripheral blood mononuclear cells was used to identify genomic alterations, using the FoundationOne® Heme test (Cambridge, MA).

### Statistical analyses

Baseline demographics, disease characteristics, and response outcomes are reported descriptively. OS and EFS were estimated using Kaplan–Meier methods. Differences in the levels of 2-HG between response groups were compared using unpaired *t*-test. Relationships between co-mutation burden at baseline and response status were evaluated by Student’s t-test or Fisher’s exact test, as appropriate.

## Results

**Fig. 1:**
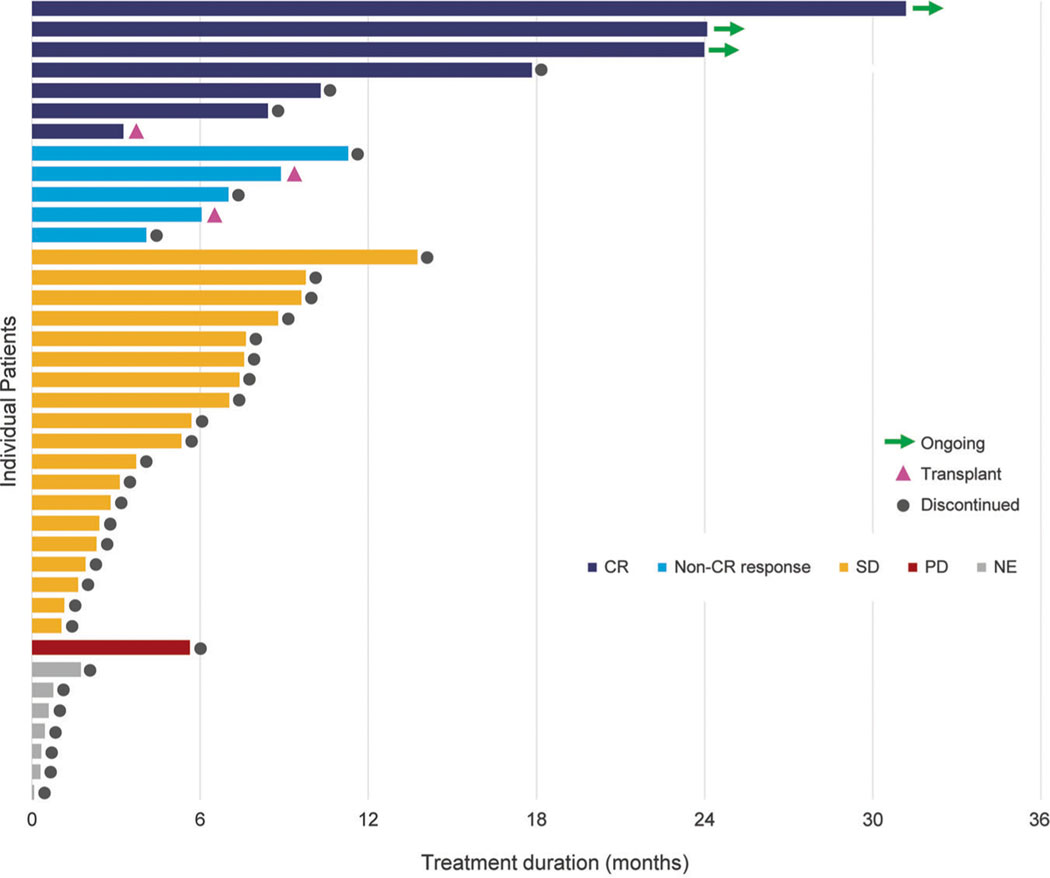
Treatment durations, hematologic responses and patient dispositions

In all, 39 patients with newly diagnosed AML were enrolled. Median follow-up duration was 8.4 months. At data cutoff (September 1, 2017), three patients, each of whom achieved a CR during enasidenib therapy, remained on-study: two patients had completed 27 treatment cycles and one patient had completed 35 cycles (Fig. 1). The median number of enasidenib treatment cycles received by all patients was 6.0 (range 1–35). Ten patients were enrolled in the dose-escalation phase of the study: one received enasidenib 50 mg once daily and nine patients received daily doses >100 mg (150–450 mg); the remaining 29 patients were treated with the recommended 100 mg daily dose in the study extension phase. Reasons for treatment discontinuation were: disease progression (*n* = 13), death (*n* = 5), withdrawal of consent (*n* = 4), investigator decision (*n* = 4), adverse event (*n* = 4), bone marrow transplant (*n* = 3), protocol violation (*n* = 2), and treatment failure (*n* = 1).

At study entry, median age was 77 years (range 58–87) and median time from AML diagnosis was 1.0 month (range 0.1–4.7) (Table 1). Twenty-six patients (67%) had *ECOG* Eastern Cooperative Oncology Group, *ELN* European LeukemiaNet, *RBC* red blood cells, *WBC* white blood cells an *IDH2*-R140 mutation and 12 patients (31%) had an *IDH2*-R172 mutation (mutant *IDH2* allele information was missing for one patient). Ten patients (26%) had National Comprehensive Cancer Network (NCCN)-defined poor-risk cytogenetics [20]. Twenty-three patients (59%) had had an antecedent hematologic disorder, including 17 patients with a prior diagnosis of myelodysplastic syndromes (MDS), three with chronic myelomonocytic leukemia (CMML), two with myelofibrosis (MF), and one patient with polycythemia vera (PCV).

**Fig. 2:**
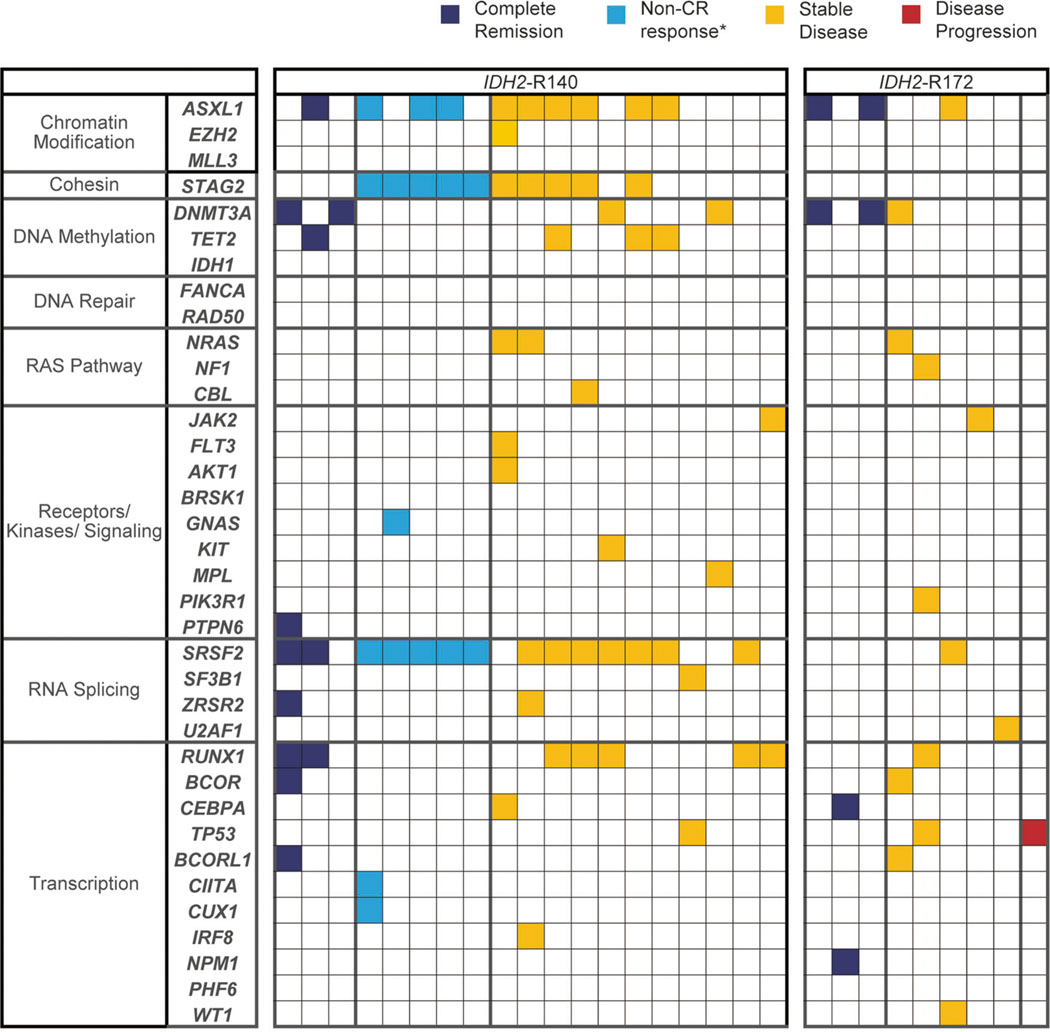
Baseline gene co-mutations, organized by functional category, *IDH2* mutation, and clinical response status

Baseline co-mutation data (i.e., mutations in addition to *IDH2*) were available for 34 patients (87%; Supplementary Fig. 1). Genes mutated in ≥10% of patients were *SRSF2* (53%), *ASXL1* (50%), *STAG2* (35%), *RUNX1* (29%), *DNMT3A* (24%), *TET2* (15%), and *NRAS* (12%) (Fig. 2). Co-mutation burden was significantly higher in the subgroup of patients with *IDH2*-R140 mutations *versus* patients with *IDH2*-R172 mutations (mean 3.9 *versus* 2.3, respectively; *P* = 0.0088) (Supplementary Fig. 2).

### Safety

The most common TEAEs (any grade, regardless of attribution to study drug) were fatigue (44%), decreased appetite (41%), nausea (38%), constipation (38%), hyperbilirubinemia (36%), peripheral edema (36%), and anemia (33%) (Supplementary Table 1). The most common treatment-related TEAEs were indirect hyperbilirubinemia (31%), nausea (23%), fatigue (18%), decreased appetite (18%), rash (18%), and anemia (15%) (Table 2). Treatment-related TEAEs led to enasidenib dose reductions for three patients (8%) and to treatment interruptions for nine patients (23%). Two patients (5%) discontinued enasidenib due to treatment-related TEAEs (thrombocytopenia, cardiac tamponade).

Treatment-related grade 3–4 TEAEs were reported for 19 patients (49%). The most common (>1 patient) were anemia (*n* = 5, 13%), indirect hyperbilirubinemia (*n* = 5, 13%), IDH differentiation syndrome (*n* = 4, 10%), thrombocytopenia (*n* = 3, 8%), tumor lysis syndrome (*n* = 3, 8%), leukopenia (*n* = 2, 5%) and lipase increase (*n* = 2, 5%). Treatment-related grade 3–4 cytopenias (any type) were reported for eight patients (21%); no treatment-related grade 3–4 infections were reported. The only nontreatment-related grade 3–4 infectious event to occur in >2 patients was pneumonia (*n* = 7, 18%).

Twelve treatment-related serious TEAEs were reported in eight patients (21%). They were tumor lysis syndrome (*n* = 2), and (*n* = 1 each): anemia, leukocytosis, cardiac tamponade, diastolic dysfunction, pericardial effusion, gastrointestinal hemorrhage, infection, decreased appetite, acute kidney injury, and IDH differentiation syndrome (IDH-DS).

An independent Differentiation Syndrome Review Committee (DSRC; including the authors SdB, CDD, and EMS) conducted a retrospective review of TEAEs consistent with IDH-DS (e.g., dyspnea, fever, peripheral edema, weight gain, pulmonary infiltrates, hypoxia), in the absence of secondary causes, for all patients in the study [21]. The DSRC identified five patients (13%) with previously untreated AML as likely having experienced IDH-DS. Median time to IDH-DS onset was 48 days (range 10–99) and events ranged in duration from 8 to 34 days. The most common symptoms were peripheral edema and dyspnea (*n* = 3 patients each). Two patients received corticosteroids to manage IDH-DS. Two patients had concomitant leukocytosis and were treated with hydroxyurea. Enasidenib was temporarily interrupted for four patients with suspected IDH-DS, including one patient who resumed treatment at a dose decreased from 450 mg/day to 300 mg/day. Three of the five patients with IDH-DS, with total enasidenib treatment durations of 5.3, 5.7, and 9.8 months, did not achieve a formal response but maintained stable disease for ≥56 consecutive days on study, and the other two patients discontinued prior to an efficacy evaluation. IDH-DS resolved without sequelae for four of the five patients, none of whom permanently discontinued treatment due to the event.

The only treatment-related death on study occurred in an 83-year-old female who developed pericardial effusion with increasing white blood cells and peripheral blast counts at 2 weeks after starting treatment and died due to cardiac tamponade. The patient had a “do not resuscitate” advance directive in place at the time of death and did not receive corticosteroid treatment. Retrospective review by the DSRC suggested that events leading to death may have been related to IDH-DS.

### Efficacy

Twelve patients achieved an IWG-defined response on study (ORR 30.8% [95% CI 17.0, 47.6]). Median times to first and best responses were 1.9 and 3.7 months, respectively. Seven patients (18%) attained CR and one patient (3%) had CRi. The median duration of any response was not reached (NR) (Table 3). Three patients (one in CR, one with CRp, and one with MLFS) proceeded to allogeneic stem cell transplant and were alive and in remission at 28, 32, and 38 months post transplant. ORRs were similar between patients with *IDH2*-R140 mutations and those with *IDH2*-R172 mutations (31% [8/26] and 33% [4/12], respectively). Nineteen patients (49%) did not achieve a response but maintained stable disease for ≥56 consecutive days on study.

Of the 23 patients with a documented antecedent hematologic disorder at study entry, five achieved a response, including three patients who achieved CR; 13 patients maintained stable disease at all response evaluations; and one patient experienced only disease progression. The remaining four patients were not evaluable because they discontinued before undergoing a formal response assessment.

Mean hemoglobin and platelet levels tended to rise in patients over the course of treatment (Supplementary Fig. 3). Eleven of 30 patients (37%) who were RBC transfusion-dependent at baseline achieved transfusion independence on-study, and 3 of 19 patients (16%) who were platelet transfusion-dependent at baseline became transfusion-independent. Of 16 patients who were RBC transfusion-dependent at entry and who maintained stable disease at all response assessments (i.e., had no IWG-defined response on-study), 6 (38%) attained RBC transfusion independence during treatment.

2-HG levels at baseline and on treatment were available for 23 patients. Median 2-HG levels were reduced from baseline during cycle 1 and remained low during subsequent treatment cycles, with greater reductions in the subgroup of patients with *IDH2*-R140 mutations than in patients with *IDH2*-R172 mutations (Supplementary Fig. 4). There were no significant differences in median baseline 2-HG levels or maximum on-treatment 2-HG reductions among efficacy-evaluable patients (*n* = 22) who attained a CR or a non-CR response, compared with those who had no response to treatment (*P* = 0.26 and *P* = 0.75, respectively) (Supplementary Fig. 5).

Baseline co-mutation and response data were available for 28 patients (72%). The mean number of co-mutations was 3.4 for both responders (*n* = 11) and nonresponders (*n* = 17) (*P* = 0.99; Supplementary Fig. 6A). Response rates were nominally, but non-significantly, different based on co-mutation burden at study entry: ORR was 47.1% for patients with ≤3 co-occurring mutations at baseline versus 27.3% for patients with ≥4 baseline co-mutations (*P* = 0.43; Supplementary Fig. 6B). Presence of a *DNMT3A* mutation was significantly associated with achievement of CR (*P* = 0.045); accordingly, patients with co-mutations in genes in the DNA methylation functional category showed a trend toward attainment of CR (*P* = 0.07) (Fig. 2). Unlike previous findings in patients with R/R AML treated with enasidenib [15], no association was observed between mutations in the RAS signaling pathway and decreased likelihood of response.

**Fig. 3:**
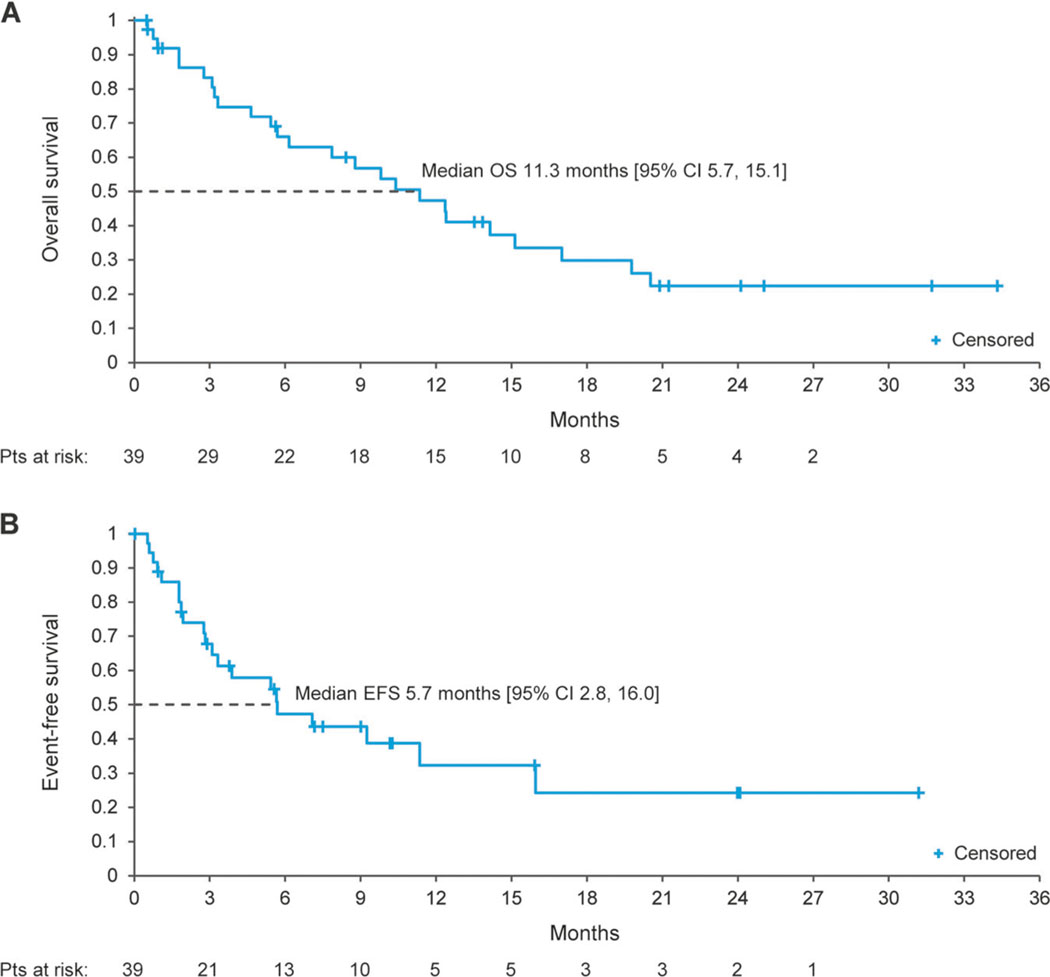
Kaplan–Meier survival estimates. **a** Overall survival (OS); and **b** event-free survival (EFS)

Estimated median OS for all patients was 11.3 months (95%CI 5.7, 15.1) (Fig. 3a). Median OS among responding patients (*n* = 12) was NR (95% CI 10.4, NR; Supplementary Fig. 7A). Median OS for the 23 patients who developed AML secondary to an antecedent hematologic disorder was 8.8 months (95%CI 3.2, 14.1; Supplementary Fig. 8). Median EFS was 5.7 months (95% CI 2.8, 16.0) (Fig. 3b). Median EFS was NR in responding patients (95%CI 9.4, NR) and was 2.8 months (1.8, 5.4) in nonresponding patients (Supplementary Fig. 7B). Thirty- and 60- day mortality rates from the start of enasidenib therapy were 8% and 13%, respectively.

## Discussion

Risk-benefit considerations are critical when choosing AML therapy options for older patients; noncytotoxic regimens are particularly important for older patients with comorbidities and adverse disease features. Enasidenib was generally well tolerated in this older population of patients with newly diagnosed AML. There was a low rate of enasidenib-related grade 3–4 cytopenias (21%) compared with what has been reported with other AML treatments [22–25], and pneumonia was the only infectious event reported for more than two patients and was not considered to be related to enasidenib. Only two patients discontinued treatment due to an enasidenib-related TEAE. Early mortality rate (13% at 60 days) was lower relative to that reported for older patients with AML who are fit enough to receive intensive chemotherapy [2]. IDH-DS occurred with similar frequency in this patient cohort (12.8%) to that reported for the 281 R/R AML patients in this study (11.7%) [21]. Differentiation syndrome reflects the mechanism of enasidenib activity as a differentiating agent. The symptoms of IDH-DS are recognizable and treatable with prompt administration of corticosteroids and other supportive care measures [21].

Patients with previously untreated AML in this study comprised a group with markedly poor clinicopathologic prognostic features, as expected given a patient population unfit for standard AML therapy, including median age of 77 years, a high rate (59%) of antecedent hematologic disorders, and a substantial proportion with poor-risk genomic features at study entry. In a multivariate analysis of data from 998 patients aged ≥65 years with AML or higher-risk MDS who received induction chemotherapy, older age, poor performance status, adverse karyotype, and prior hematologic disorders were all independently predictive of lower CR rates, increased 8-week post-induction mortality, and reduced OS [26]. Despite these adverse features, approximately one-third of our patients had a hematologic response during enasidenib therapy. Furthermore, enasidenib served as a bridge to potentially curative transplant for three patients. Responses were durable; median duration of any response was NR at a median follow-up of 8.4 months. Of patients with no IWG response who maintained stable disease during treatment, 38% achieved RBC transfusion independence, which is associated with prolonged survival and improved quality of life in AML [27].

The most common co-mutations in our patients were those known to frequently co-occur with *IDH2* mutations, including mutations in *SRSF2*, *ASXL1*, *STAG2*, and *RUNX1*; these mutations are associated with older age, secondary AML, and poor outcomes [9, 28–30]. In this newly diagnosed AML population, the presence of *DNMT3A* co-mutations was significantly associated with achievement of CR, albeit this finding is based on a small number of patients. As DNMT3A helps regulate DNA methylation [31], mutations in *DNMT3A* could cooperate with enasidenib to suppress DNA hypermethylation in patients with mutant-*IDH2*/*DNMT3A* AML. *DNMT3A* mutations are often associated with poor prognosis in AML [31, 32], but have been associated with increased likelihood of CR in patients with MDS or previously untreated AML who receive HMAs [33, 34]. *DNMT3A* co-mutations were not significantly associated with response to enasidenib monotherapy in patients with R/R AML participating in the current study.

The overall response rate in these newly diagnosed patients was marginally lower than that reported for enasidenib-treated patients with R/R AML (median age of 70 years [16]); however, median OS in the current, older, population was nearly 1 year and was NR for patients experiencing a hematologic response during enasidenib therapy. Survival outcomes in this group are promising. Initial treatment with azacitidine in older patients (age ≥65 years) with newly diagnosed AML was associated with a median OS of 10.4 months versus 6.5 months for patients treated with conventional care regimens (induction chemotherapy, low-dose cytarabine, or best supportive care) [24]. Median OS in a similar patient population treated with decitabine was 7.7 months [25].

AML secondary to MDS or other antecedent hematologic disorders such as myeloproliferative neoplasms has a particularly poor prognosis. A recent study comparing CPX-351 (Vyxeos®, Jazz Pharmaceuticals, Palo Alto, CA) with standard cytarabine plus daunorubicin (7 + 3) induction chemotherapy in patients with newly diagnosed AML secondary to a previous hematologic disorder showed the median OS in the CPX-351 and standard chemotherapy arms was 9.56 and 5.95 months, respectively [35]. In the current study, the 23 patients who developed AML after a previous diagnosis of MDS or a myeloproliferative neoplasm and who were not candidates for standard AML therapy had a median OS from the start of enasidenib therapy of 8.8 months. These results suggest enasidenib may benefit older adults with newly diagnosed mutant-*IDH2* secondary AML who are not fit to receive cytotoxic regimens. Indeed, current NCCN guidelines recommend enasidenib for treatment of patients with newly diagnosed mutant-*IDH2* AML who are not candidates for standard AML treatment [36].

Enasidenib is under investigation in this patient population in the BEAT AML Master trial (ClinicalTrials.gov NCT03013998). Because it is noncytotoxic and generally well tolerated with minimal drug interactions, use of enasidenib in combination with other AML treatments may prove beneficial and is under investigation in patients with newly diagnosed mutant-*IDH2* AML in combination with azacitidine (ClinicalTrials.gov NCT02677922), and in combination with standard induction chemotherapy (ClinicalTrials.gov NCT02632708).

## Supplementary Material

1571616_Sup_Material

## Figures and Tables

**Table 1:** Demographic and disease characteristics at baseline

	Patients with newly diagnosed AML *N* = 39

Age (years), median (range)	77 (58–87)
Age ≥75 years, *n*(%)	24 (62)
Sex, *n*(%)	
Male	28 (72)
Female	11 (28)
AML classification,* *n*(%)	
Myelodysplasia-related changes	14 (36)
Recurrent genetic abnormalities	2(5)
Therapy-related myeloid neoplasms	2(5)
Not otherwise specified	20 (51)
Missing	1 (3)
Months since initial diagnosis, median (range)	1.0 (0.1–4.7)
Antecedent hematologic disorder,^a^ *n*(%)	23 (59)
Cytogenetic risk status, *n*(%)	
Intermediate-risk	19 (49)
Poor-risk	10 (26)
Missing	9 (23)
ECOG performance status score, *n*(%)	
0	12 (31)
1	18 (46)
2	9 (23)
*IDH2* mutant allele, *n*(%)	
R140	26 (67)
R172	12 (31)
Other/Missing	1 (3)
ELN risk classification, *n*(%)	
Favorable	1 (3)
Intermediate	9 (23)
Adverse	25 (64)
Not assessable	4 (10
Bone marrow blasts (%),^b^ median (range)	39 (14–92)
Hematology, mean (SD)	
WBC (10^9^/L)	5.8 (7.0)
Hemoglobin (g/dL)	9.1 (10.4)
Platelet (10^9^/L)	89 (92)
Transfusion dependent, *n*(%)	
RBC	30 (77)
Platelets	19 (49)

* per World Health Organization (WHO) 2008 AML classifications of myeloid neoplasms

a Myelodysplastic syndromes or myeloproliferative neoplasms (chronic myelomonocytic leukemia, myelofibrosis, or polycythemia vera)

b Local assessment

**Table 2:** Enasidenib-related adverse events reported in ≥10% of patients

Adverse event	Patients with newly diagnosed AML *N* = 39	
	
	All grades *n* (%)	Grade ≥3

Hyperbilirubinemia	12 (31)	5 (13)
Nausea	9 (23)	0
Decreased appetite	7 (18)	1 (3)
Fatigue	7 (18)	1 (3)
Thrombocytopenia	7 (18)	3 (8)
Rash	7 (18)	0
Anemia	6 (15)	5 (13)
IDH differentiation syndrome	5 (13)	4 (10)^a^
Dysgeusia	4 (10)	0
Electrocardiogram QT prolongation	4 (10)	1 (3)
Peripheral neuropathy	4 (10)	0
Tumor lysis syndrome	4 (10)	3 (8)
Vomiting	4 (10)	0

a The only treatment-related grade 5 event was cardiac tamponade, which was retrospectively adjudicated by the Differentiation Syndrome Review Committee to have potentially been related to IDH differentiation syndrome

**Table 3:** Hematologic responses, times to response, and durations of response

	Patients with newly diagnosed AML *N* = 39

Overall response rate (ORR),^a^ *n*(%)	**30.8% (12/39)**
95% CI	17.0, 47.6
Best response, *n*(%)	
Complete remission (CR)	7 (18)
CR with incomplete count recovery (CRi/CRp)	1 (3)
Partial remission	2 (5)
Morphologic leukemia-free state	2 (5)
Stable disease,^b^ *n*(%)	19 (49)
Disease progression, *n*(%)	1 (3)
Not evaluable,^c^ *n*(%)	7 (18)
Time to first response, months, median (range)	1.9 (1.0–3.8)
Time to best response, months, median (range)	3.7 (1.0–12.9)
Duration of any response, months, median [95% CI]	NR [7.4, NR]
Time to CR, months, median (range)	5.6 (3.4–12.9)
Duration of CR, months, median [95% CI]	NR [3.7, NR]

a Overall response included complete remission (CR), CR with incomplete count recovery, partial remission, and morphologic leukemia-free state, per modified IWG 2003 response criteria for AML

b Failure to achieve a response but not meeting criteria for disease progression, sustained for a period of ≥8 weeks

c Patients discontinued before undergoing a clinical response assessment

95% CI, 95% confidence interval; *NR* not reached
