# Supplementary material for: Enasidenib, an inhibitor of mutant IDH2 proteins, induces durable remissions in older patients with newly diagnosed acute myeloid leukemia
Source: Leukemia. Author manuscript; Available in PMC 2022 Dec 6. (PMC9724489; doi:10.1038/s41375-019-0472-2)
Supplement: 1571616_Sup_Material [file NIHMS1571616-supplement-1571616_Sup_Material.pdf]

Supplementary Material

Supplementary Table 1. Treatment-emergent adverse events (any grade, any cause) reported in >20% of patients

Adverse event	Patients with Newly Diagnosed AML N=39 n (%)
Fatigue	17 (44)
Decreased appetite	16 (41)
Constipation	15 (38)
Nausea	15 (38)
Hyperbilirubinemia	13 (33)
Peripheral edema	14 (36)
Anemia	13 (33)
Diarrhea	11 (28)
Dyspnea	11 (28)
Vomiting	10 (26)
Cough	9 (23)
Headache	9 (23)
Hypokalemia	9 (23)
Acute kidney injury	8 (21)
Dizziness	8 (21)
Hypocalcemia	8 (21)
Pneumonia	8 (21)
Thrombocytopenia	8 (21)

Supplementary Figure 1. Baseline co-mutations in patients with untreated AML

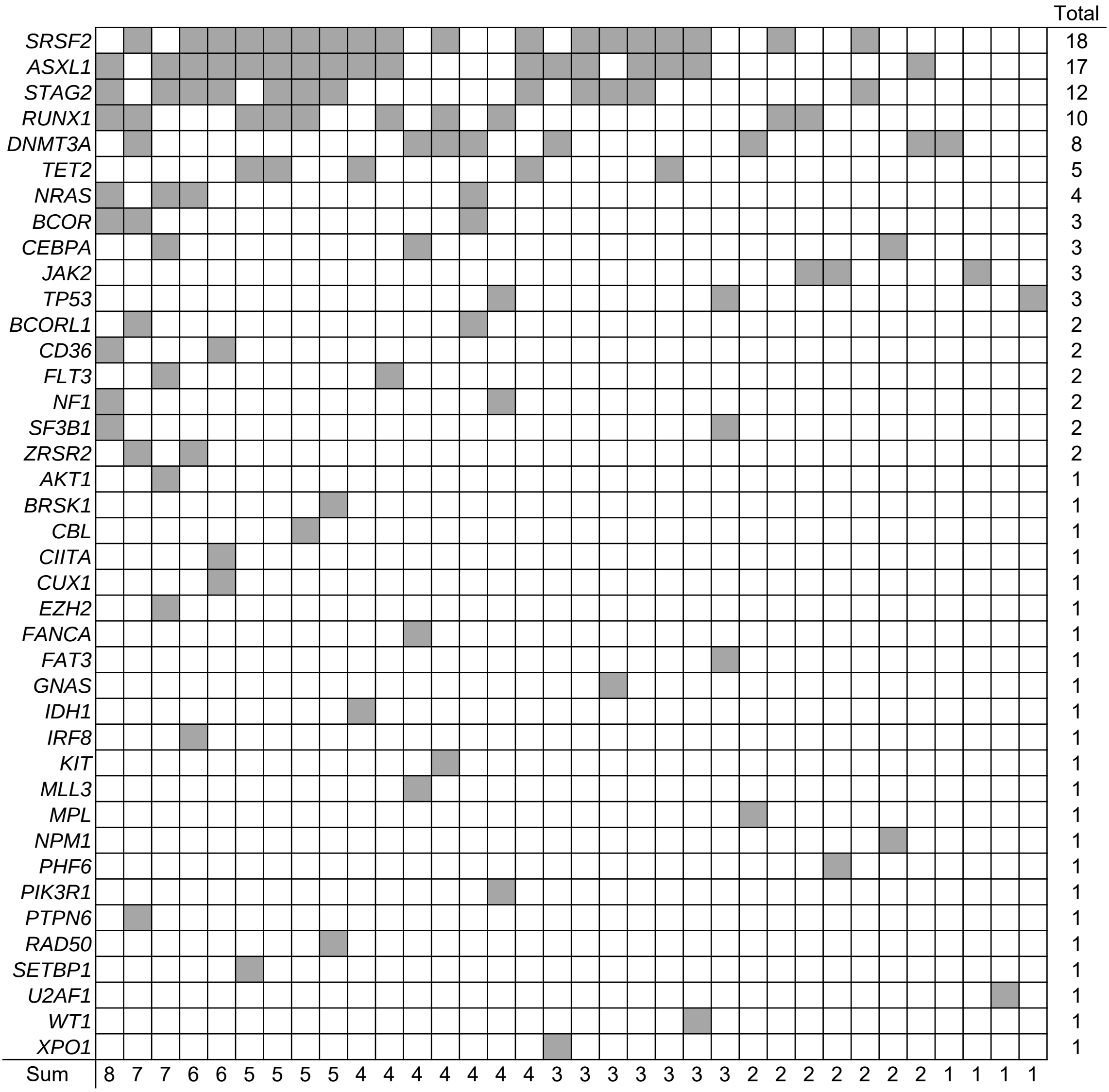

Supplementary Figure 2. Co-mutation burden by IDH2 mutant allele. Horizontal line and error bars represent the mean ± SD. P value was calculated using Student's t-test.

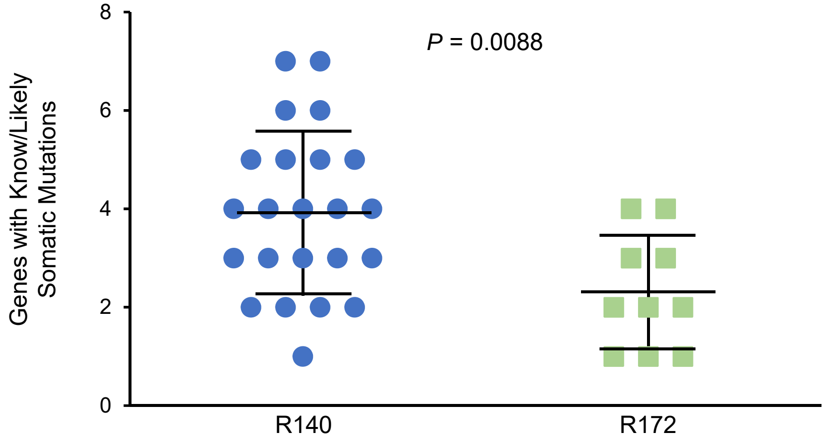

	R140	R172
N patients	23	10
Mean number of co-mutations	3.9	2.3

Supplementary Figure 3. Mean hematology counts during treatment cycles 1 through 18

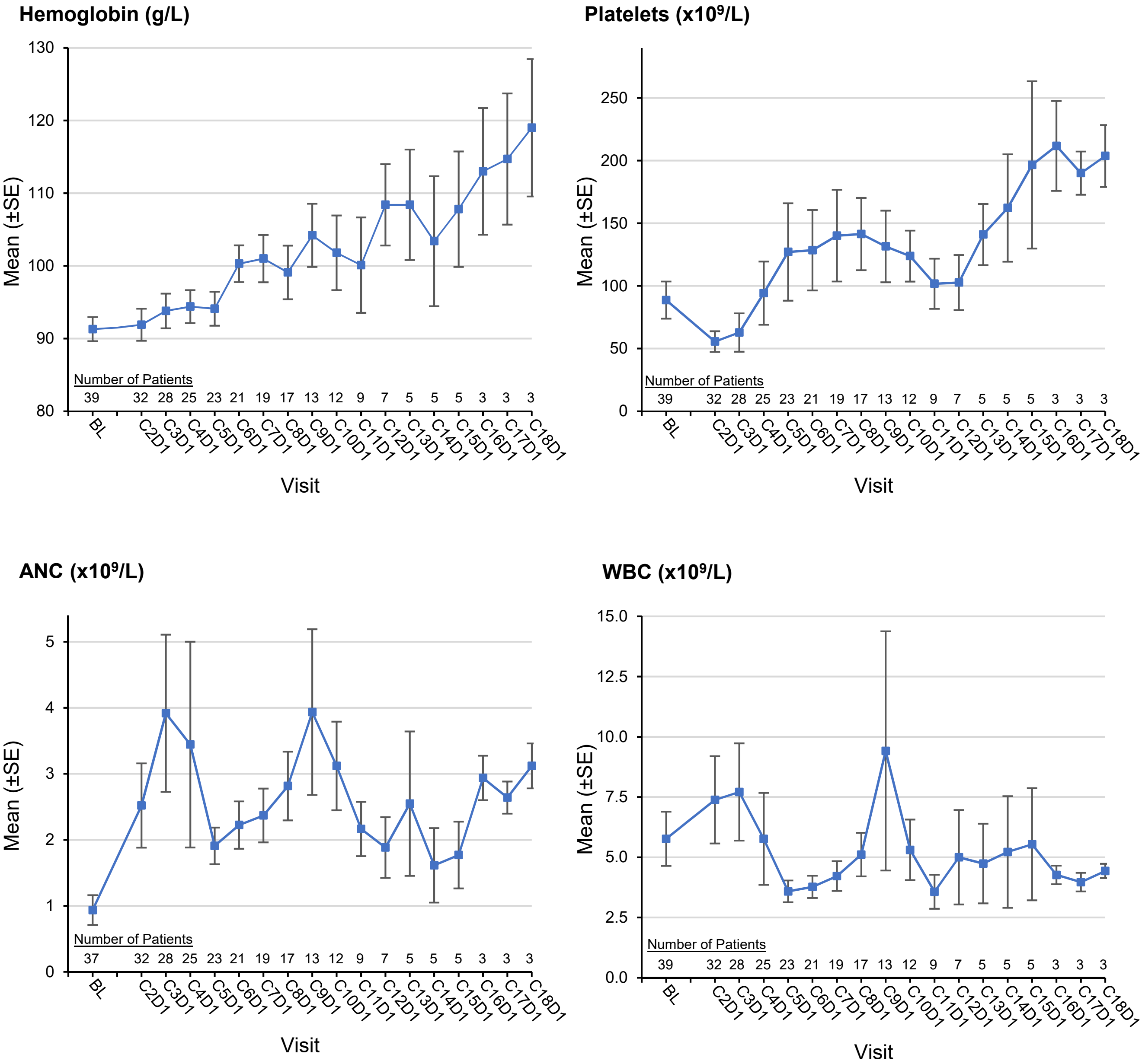

Supplementary Figure 4. Baseline-normalized 2-HG concentrations during enasidenib treatment (n=23). A) Patients with IDH2-R140 mutations (n=15). **B)** Patients with IDH2-R172 mutations (n=8). Dotted lines indicate normalized baseline 2-HG concentrations

A

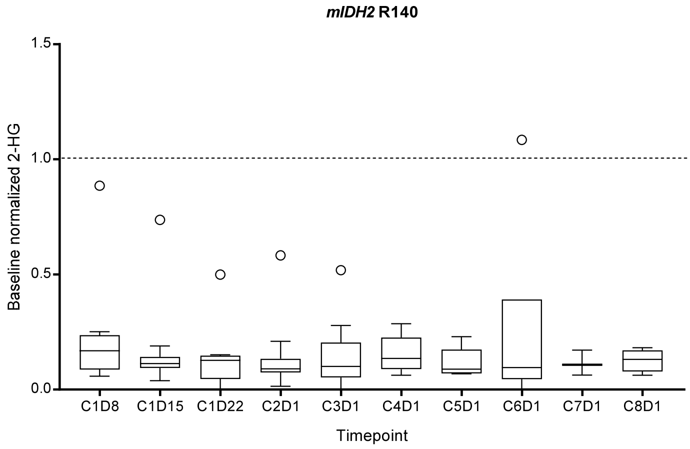

Cycle	1			2	3	4	5	6	7	8
Day	8	15	22	1	1	1	1	1	1	1
N patients	10	11	9	14	9	8	5	6	3	4
Median	0.167	0.113	0.127	0.091	0.100	0.135	0.089	0.096	0.108	0.131

B

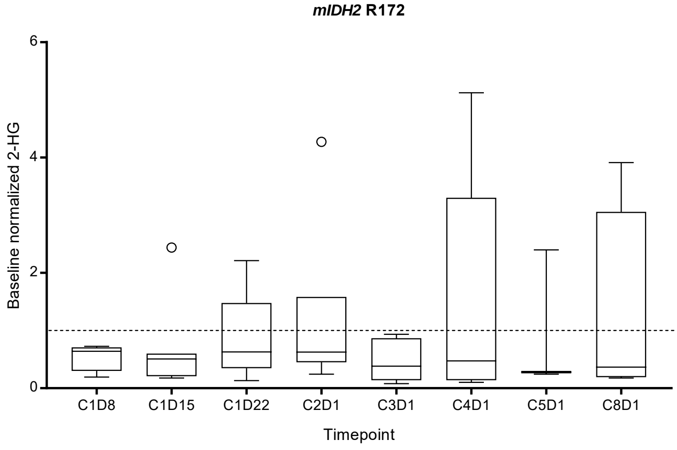

Cycle	1			2	3	4	5	8
Day	8	15	22	1	1	1	1	1
N patients	4	7	5	6	5	5	3	4
Median	0.638	0.505	0.629	0.624	0.383	0.471	0.277	0.366

Supplementary Figure 5. 2-HG concentrations at baseline and on-treatment 2-HG suppression, by clinical response status in efficacy-evaluable patients (n=22). A) 2-HG levels at baseline by on-study response category. **B)** Baseline-normalized minimum 2-HG levels during treatment by response status. Dotted line indicates normalized baseline 2-HG concentration. Responders include patients with complete remission (CR), CR with incomplete hematologic or platelet recovery (CRi/CRp), partial remisison, or morphologic leukemia-free state. Non-response includes stable disease and progressive disease.

A

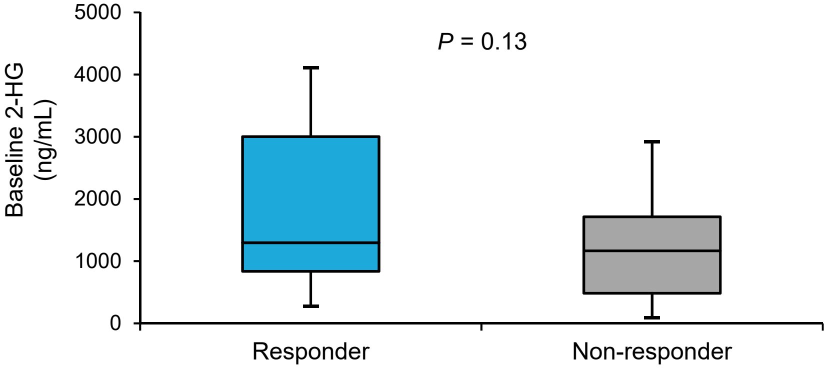

N patients	8	14
Median 2-HG	1297	1167

B

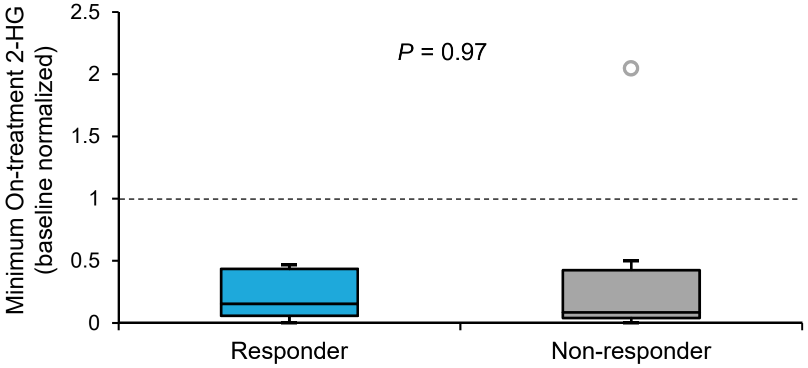

N patients	7	15
Median 2-HG	0.154	0.086

Supplementary Figure 6. Co-mutation burden at baseline and clinical response status (n=28). **A)** Number of baseline co-mutations in responding and non-responding patients. Horizontal line and error bars represent mean ± standard deviation. **B)** Overall response rate (ORR) in patients with ≤3 vs ≥4 co-mutations at baseline. Responses include complete remission (CR), CR with incomplete hematologic or platelet recovery (CRi/CRp), partial remission (PR), and morphologic leukemia-free state (MLFS). Non-responses include stable disease (SD) and progressive disease (PD).

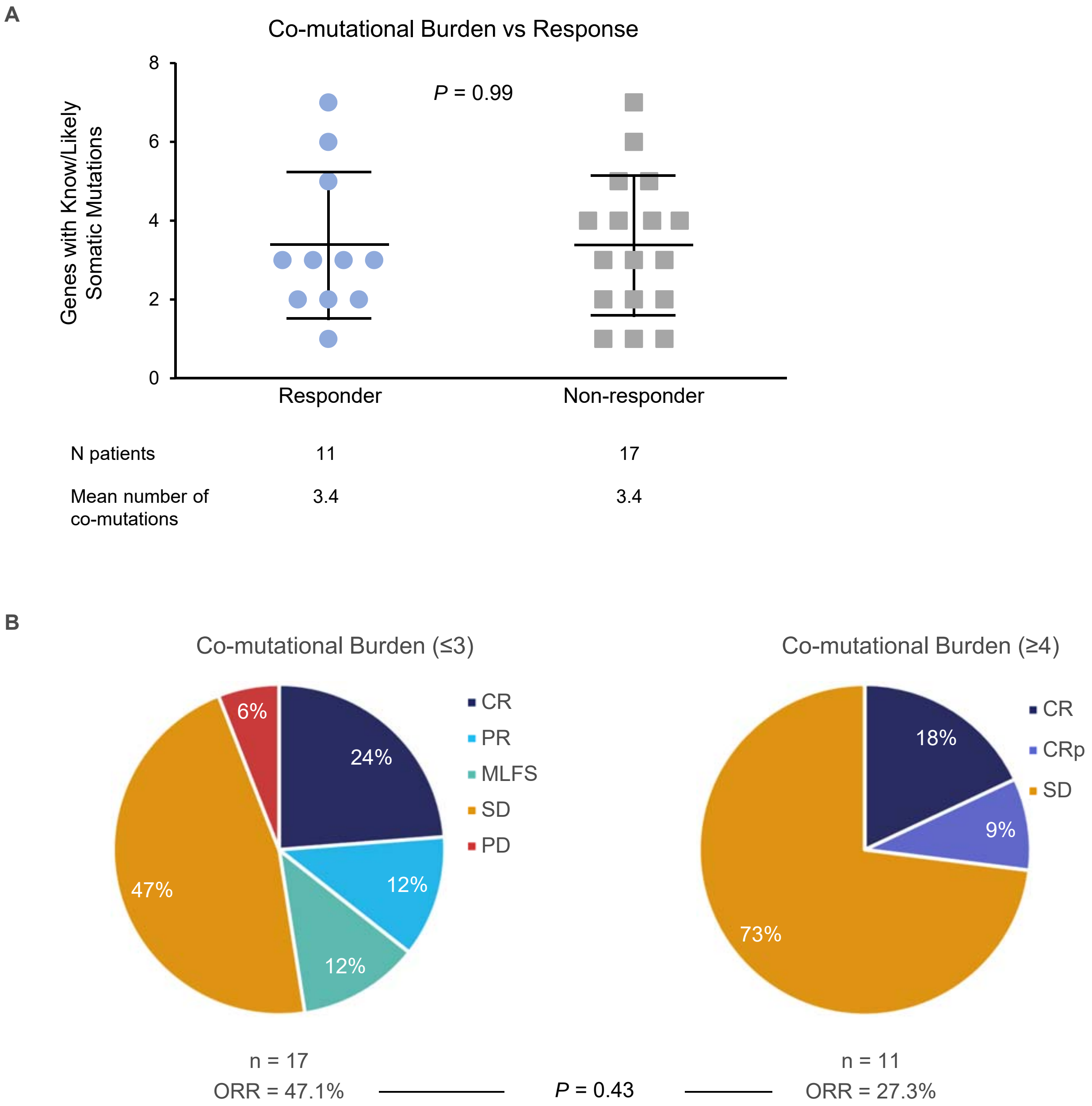

Supplementary Figure 7. Survival estimates by clinical response status. A) Overall survival; **B)** Event-free survival

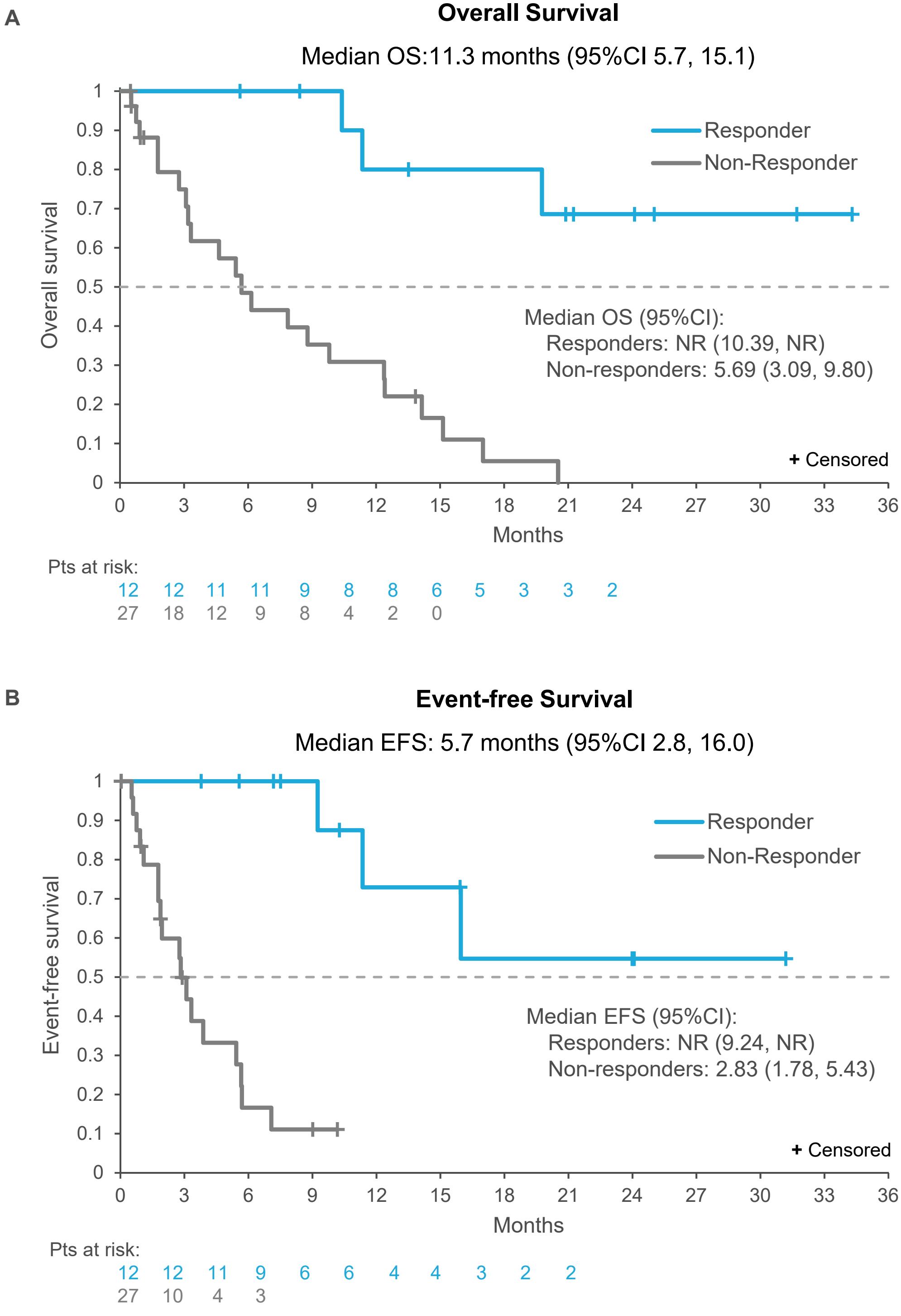

95%CI, 95% confidence interval; EFS, event-free survival; NR, not reached; OS, overall survival

Supplementary Figure 8. Estimated overall survival among patients with a known antecedent hematologic disorder* (N=23)

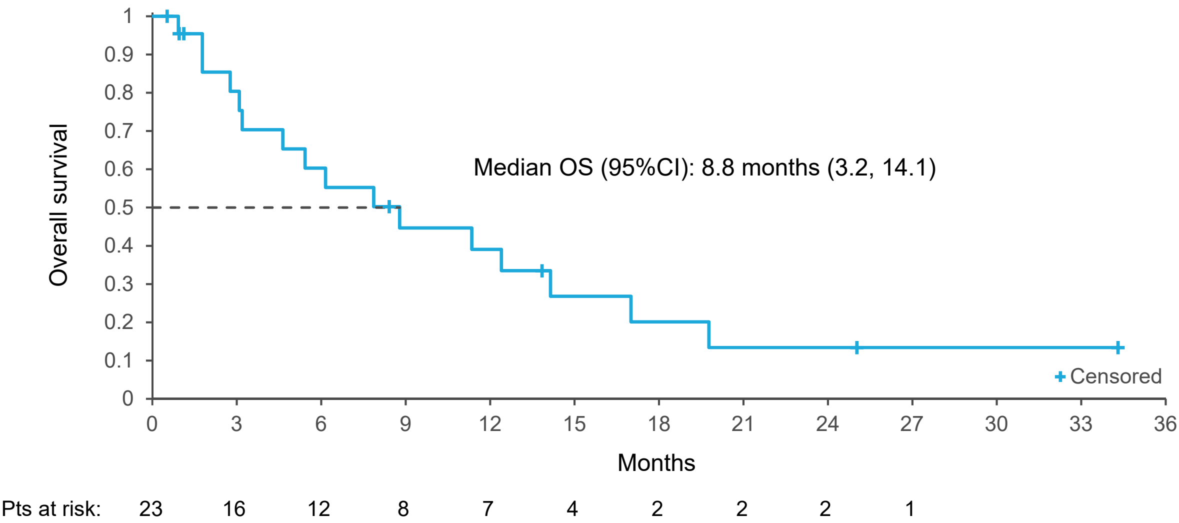

*Includes myelodysplastic syndromes (n=17), chronic myelomonocytic leukemia (n=3), myelofibrosis (n=2), or polycythemia vera (n=1).
95%CI, 95% confidence interval; OS, overall survival
